# 快速康复外科在肺癌手术中的应用研究

**DOI:** 10.3779/j.issn.1009-3419.2010.02.04

**Published:** 2010-02-20

**Authors:** 光强 赵, 云超 黄, 小波 陈, 林灿 段, 千里 马, 玉洁 雷, 凯云 杨, 霁阳 王

**Affiliations:** 650118 昆明, 昆明医学院第三附属医院(云南省肿瘤医院)胸心外科 Department of Cardiothoracic Surgery, the Third Affiliated Hospital of Kunming Medical College (The Tumor Hospital of Yunnan Province), Kunming 650118, China

**Keywords:** 快速康复外科, 手术治疗, 肺肿瘤, Fast track surgery, Surgical treatment, Lung neoplasms

## Abstract

**背景与目的:**

快速康复外科(fast track surgery, FTS)通过减少手术病人生理和心理的创伤应激, 从而达到病人快速康复的目的。本研究旨在探讨FTS技术在肺癌手术中应用的可行性。

**方法:**

选取80例适合单叶肺叶切除手术治疗的肺癌患者, 随机分为实验组及对照组, 实验组接受FTS方案治疗、对照组接受传统方法治疗。比较两组术后不同时间的疼痛程度、肺不张发生率、胸腔积液发生率、术后住院时间及住院总费用。

**结果:**

FTS方案组:术后1 h、6 h、12 h、24 h、48 h疼痛VAS评分均明显低于传统治疗组; 肺不张发生率为10.53%, 胸腔积液发生率为26.31%, 术后住院时间(4±1)d, 住院总费用(1.56 ±0.76)万元。对照组:肺不张发生率为33.33%, 胸腔积液发生率为22.22%, 术后住院时间(9±1)d, 总费用(2.36 ±0.54)万元。两组比较:术后疼痛程度(视觉模拟评分)明显低于对照组, 差异有统计学意义(*P* < 0.01);肺不张发生率(*P*=0.035), 住院时间(*P*=0.021)及住院总费用(*P*=0.024)的组间差异均有统计学意义; 胸腔积液发生率的组间差异无统计学意义(*P*=0.223)。

**结论:**

FTS方案的应用可有效促进肺癌患者术后的康复, 减少术后并发症发生, 缩短住院时间, 降低住院费用。

## Abstract (en)

**Background and objective:**

Fast track surgery (FTS) is a systematical method to accelerate the recovery of surgical patients by reducing the physical and mental trauma stress of them.The research is to investigate the feasibility of FTS application in lung cancer surgery.

**Methods:**

A total of 80 cases of lung cancer patients with single leaf lobotomy resection were randomized into two groups.While the experimental group was treated with the conception of FTS, and the control group was treated with the traditional methods.The incident rate of post-operation pain degrees, telecasts, pleural effusion, the post-operation time stay in hospital time and the total cost during hospitalization in two groups were compared respectively.

**Results:**

In FTS group:the VAS score of post-operation pain at 1 h, 6 h, 12 h, 24 h and 48 h all significantly decreased compared to the traditional therapy group.The incidence rate of telecast was 10.53%.The incidence rate of pleural effusion was 26.31%.The length of stay after operation was (4±1) d and the total cost was RMB 15 600±7 600.In the control group, the above values were 77.78%, 33.33%, 22.22%, (9±1) d, RMB 23 600±5 400, respectively.The post operation pain (VAS method) of FTS group was remarkablely below the control group.There has significant difference of the incident rate of telecasts, stay time in hospital and the total cast in two groups (*P* < 0.05).No significant difference was observed in the incident rate of pleural effusion.

**Conclusion:**

The new methods of FTS can apparently accelerates recovery after lung cancer resection, reduces complications, shorten timestay in hospital and cut down the total cost.

快速康复外科(fast track surgery, FTS)是指采用一系列有循证医学证据的围手术期处理的优化措施, 它能减少手术病人生理和心理的创伤应激, 从而达到病人快速康复的目的^[1-3]^。其核心是减少病人的创伤和应激损害。它是在多学科协助治疗下产生的最佳结果。FTS在结直肠手术切除等许多外科疾病中获得成功应用^[4-6]^, 但尚未在肺癌手术中广泛应用。我国是世界肺癌高发区之一, 肺癌患者数量居世界第一位, 如何将该技术合理应用到肺癌外科治疗中对我国肺癌患者具有重要意义。我们对其在肺癌手术中的应用可行性进行了初步探讨, 现将治疗体会报道如下。

## 1 材料与方法

### 1.1 一般资料

选取昆明医学院第三附属医院(云南省肿瘤医院)2008年6月-2009年6月肺癌手术患者80例。入选标准:术前均未接受放疗或化疗, 术前估计可以行单叶肺叶切除术、无糖尿病史、患者同意FTS治疗。排除标准:有严重器官功能障碍、姑息手术或多叶肺叶切除术的患者。随机分为FTS组和对照组, 各组病例的数据均经均衡性检验(χ^2^检验), 差异无统计学意义(*P* > 0.05), 具备可比性。具体资料见表 1。

**1:** 快速康复外科治疗组和传统治疗组肺癌病人的临床特征 Clinical characteristics of lung cancer patients in Fast track surgery group and Traditional therapy group

Clinical characteristics		Fast track surgery group (*n*=38)	Traditional therapy group (*n*=36)	*P*
Gender (*n*)	Male	24	25	0.568
	Female	14	11	
Age (year)	Range	38-70	44-72	0.334
	Median age	53.20±8.80	55.32±7.81	
Tissue type (*n*)	Squamous carcinoma	22	20	0.894
	Adenocarcinoma	13	12	
	Other type	3	4	
TNM stage (*n*)	Ⅰ	5	4	0.883
	Ⅱ	20	21	
	Ⅲa	13	11	

### 1.2 术前教育

收集患者的相关资料, 与患者及家属交谈, 了解其心理状态、性格特征以及对疾病认识的程度, 针对性地进行心理疏导。邀请治疗成功的肺癌患者做现场报告, 解除患者心理障碍, 使其积极配合手术治疗和护理。由于FTS治疗的围手术期与传统的处理措施有很大不同, 因此, 我们对接受FTS治疗的肺癌患者和家属详细介绍治疗方法、目的和意义, 说明围手术期采用一系列新方法的步骤、各阶段所需的时间等。

### 1.3 术前饮食

FTS组术前1天正常进食, 术前晚21:00再进流食300 mL-500 mL, 术晨6:00饮碳水化合物饮料300 mL-500 mL。对照组术前1天中午正常进食, 晚餐进流食, 术前晚24:00后禁饮禁食。

### 1.4 术中处理

FTS组采用全麻方法, 麻醉诱导前常规应用地塞米松10 mg, 镇静及镇痛药选用短效药物丙泊酚、瑞芬太尼。术中控制输液速度, 补液量≤1 000 mL, 根据心率和血压情况使用血管活性药物多巴胺和β受体阻滞剂艾司洛尔。注意保温, 使用输液加温器加温输注液体等措施, 使患者术中体温保持在36 ℃左右^[7]^。手术切口采用后外侧小切口开胸, 尽量少切断胸壁肌肉, 切口采用4-0可吸收缝线皮内缝合。肺裂处理全部采用一次性直线切割缝合器, 支气管残端采用一次性吻合器进行吻合。关胸前将切口上下缘肋间神经游离后进行肋间神经冷冻(冷冻气源采用液态CO_2_, 探头的工作温度为-70 ℃–-50 ℃, 冷冻持续时间为90 s), 减轻手术后切口疼痛。对照组按传统方法进行, 术后采用静脉镇痛泵镇痛(泵内药物:芬太尼20 mg, 咪唑安定10 mg, 维持48 h)。

### 1.5 术后处理

FTS组术后6 h拔除导尿管, 术后观察胸腔引流液量若有减少趋势, 则24 h内拔除引流管。手术当日患者在床上坐起1次-2次, 活动四肢尤其是下肢, 术后第1天增加床上活动量, 胸管拔除后下床活动1次-2次, 第2天开始渐增多下床活动次数。术后注意保温, 静脉补液使用输液加温器加温。术后第1-2天分别静脉冲击给予地塞米松10 mg、每日一次。术后静脉注射托烷司琼5 mg、每日一次。术后2 h观察无恶心、呕吐, 饮碳水化合物饮料300 mL, 术后4 h进流质饮食。术后第1天清晨正常饮食。术后每日控制静脉补液量在500 mL-1 000 mL。对照组按传统方法进行。

### 1.6 观察指标

快速康复外科组和传统治疗组手术后胸部疼痛的评价采用视觉模拟评分法(visual analogue scales, VAS)。0分为无痛, 1分-2分为偶尔有轻微疼痛, 3分-4分为经常有轻微疼痛, 5分-6分为偶尔有明显疼痛但可以忍受, 7分-8分为经常有明显疼痛且不能忍受, 需要口服镇痛药物, 9分-10分为剧烈疼痛, 需要注射镇痛药物。术后第1天复查胸片记录肺不张发生情况; 胸管拔除后2天复查胸片观察记录胸腔积液情况; 计算住院总费用。

### 1.7 统计学处理

采用SPSS 13.0软件分析数据。术后肺不张、拔管后胸腔积液发生率的比较采用*χ*^2^检验, 疼痛程度VAS评分、住院时间、住院费用的比较采用*t*检验。以*P* < 0.05为差异有统计学意义。

## 2 结果

FTS组和对照组分别有2例和4例术中需行两叶肺叶切除而被剔除。快速康复外科组和传统治疗组术后疼痛程度情况比较见表 2。肺不张、胸腔积液发生情况、住院时间及住院总费用的比较见表 3。

**2:** 快速康复外科治疗组和传统治疗组术后疼痛VAS评分比较 Pain degree comparisons of Fast track surgery group and Traditional therapy group

Groups	1 h	6 h	12 h	24 h	48 h
Fast track surgery group (*n*=38)	1.80±1.13	1.91±0.98	1.73±0.95	1.68±0.65	1.69±0.90
Traditional therapy group (*n*=36)	7.00±1.56	7.41±0.93	6.82±1.12	6. 65±0.76	6.34±0.88
*P*	0.002	0.005	0.010	0.023	0.031

**3:** 快速康复外科治疗组和传统治疗组肺不张、胸腔积液发生情况、住院时间经及住院总费用的比较 Comparisons of telecasts, pleural effusion, total cast stay in hospital of Fast track surgery group and Traditional therapy group

Items	Fast track surgery group (*n*=38)	Traditional therapy group (*n*=36)	*P*
Telecasts (*n*)	4	12	0.035
Pleural effusion (*n*)	10	8	0.223
Time stay in hospital (d)	4±1	9±1	0.021
Total cost stay in hospital (RMB)	15 600 ±7 600	23 600 ±5 400	0.024

FTS组肺不张发生率为10.53%, 低于传统治疗组(33.33%), 差异有统计学意义(*P* < 0.05);而FTS组胸腔积液发生率为26.31%, 与传统治疗组(22.22%)相比差异无统计学意义(*P* > 0.05);FTS组术后住院时间[(4±1)d], 住院总费用[(1.56 ±0.76)万元]均低于传统治疗组, 差异有统计学意义(*P* < 0.05)。

## 3 讨论

FTS的内容涉及多学科领域, 并非外科学的独立分支, 而是对传统外科学的重要补充与完善。FTS是一种新的外科理念, 其实质是利用现有手段将围术期各种常规治疗措施进行优化、组合, 正被广泛应用于外科各领域。经循证医学证实FTS在降低结直肠术后并发症的发生率、加快术后恢复、缩短住院时间、减少医疗费用等方面较传统方法有明显的优势。FTS在胃食管等外科领域也被证实安全性和有效性, 我国是肺癌高发区, 虽然肺癌外科手术不涉及肠道准备, 但同样面临如何快速康复问题, 这样可以使我国有限的医疗卫生资源可以得到更好利用。

本研究发现:FTS组患者, 经过医生、护士的术前宣教, 发生术前紧张血压升高、手术后因疼痛不敢咳嗽, 或者咳嗽方法错误、不敢下床活动等较传统治疗组患者少, 并且与医生、护士配合较传统治疗组患者好。

为避免麻醉时气管插管引起肺部误吸的危险, 传统方法是在择期手术的前夜即开始禁食。然而, 最近研究发现并无证据支持这一措施。近年研究^[8]^证明, 在胃功能正常的情况下, 进固体食物6 h后胃可排空, 液体2 h内即可排空。而隔夜禁食后手术, 对机体是一个很大的消耗和很强的应激, 极大地扰乱了机体内稳定。许多国家的麻醉学会现已推荐麻醉开始前2 h禁食清流质, 麻醉前6 h禁食固体饮食^[9]^。本研究发现:术前1 d正常进食, 21:00再进流食300 mL-500 mL, 术晨6:00饮碳水化合物饮料300 mL-500 mL可减少患者术前口渴、饥饿及烦躁症状。并可以显著减少术后胰岛素抵抗的发生率^[10]^, 防止饥饿引起的应激代谢。

有证据^[11]^表明, 术中和术后应大量输液以维持理想血压。但术后应激导致抗利尿激素分泌增加, 机体容易出现水钠潴溜, 如再大量输液将加重心血管负担, 延缓术后胃肠道功能的恢复, 增加肠麻痹的时间, 且可能增加术后并发症发生及延长住院日。我们采用术前不长时间禁食、不常规肠道准备及术前口服碳水化合物饮品等措施, 减少了术中输液量及盐分输入量, 减少了心律失常, 加快了患者的胃肠功能恢复, 减少了住院时间、住院费用和并发症。

手术过程中, 手术室的温度经常维持在20 ℃-25 ℃之间。患者在手术过程中始终处于裸露状态, 再加上麻醉对中枢和外周体温调节机制的干扰, 如果没有充分的保温措施, 可使患者在手术结束后的体温比正常体温大约低1 ℃-3 ℃^[12]^。有研究^[13]^表明, 术中低温可导致一系列不良的后果, 诸如使术后伤口感染率升高2倍-3倍, 增加术中失血量, 增加术中、术后心血管并发症的发生率等, 进而延缓患者的术后恢复。另外, 糖皮质激素的应用可以减少恶心、呕吐和疼痛, 减轻炎性反应。我们手术中注意保温、使用输液加温器加温输注液体等措施, 使患者术中体温保持在36 ℃左右。手术切口采用后外侧小切口开胸, 不断肋骨, 减少肌肉损伤, 肺裂处理全部采用一次性直线切割缝合器, 缩短了手术时间并增加了手术安全性, 尽可能减轻了手术对患者造成的创伤和应激反应, 促进了患者术后快速恢复。

术后疼痛是最常见的一种症状。疼痛所产生的一系列病理生理变化会对病人的康复产生不利的影响。疼痛可引起很强的应激反应, 促使体内释放出多种激素, 如儿茶酚胺、皮质激素、醛固酮等, 造成血糖升高和负氮平衡。疼痛可兴奋交感神经, 血中儿茶酚胺和血管紧张素升高, 使病人血压升高、心动过速和心律失常。胸部手术后疼痛, 还会对呼吸产生明显影响, 通气量减少, CO_2_潴留增加。

全麻气管插管术后, 气管内痰液分泌潴留增加, 切口和引流管口的疼痛抑制患者咳痰动作, 增加肺不张和肺部感染的发生率。术后静脉镇痛泵是临床上最为常用的镇痛方法, 它是通过静脉注入止痛剂, 但是也具有一些自身不可避免的缺点。主要是持续时间短, 用药剂量大, 易引起恶心、呕吐, 长时间使用易成瘾, 剂量大可引起呼吸抑制, 影响呼吸功能。同时, 大部分镇痛剂均具有镇静作用, 会影响术后患者的咳嗽、咳痰效果, 从而容易造成肺部感染、肺不张等并发症, 延长患者住院时间。FTS方案强调“有效止痛”。我们术中进行肋间神经冷冻阻滞, 不但可以达到很好的止痛效果, 有效减少应激反应, 而且减少了阿片类物质的应用, 有利于胃肠功能恢复和身体的康复, 减少术后肺部并发症和其它并发症的发生。减轻病人术后的疼痛, 不仅可减少创伤的应激反应, 而且还为病人术后早期下床活动创造条件。肋间神经冷冻阻滞所引起的病理变化是可逆的, 不会造成肋间神经永久性损害^[14]^。

传统方法胸部引流管的拔除指征是, 24 h引流量小于50 mL, 导致胸引管留置时间过长, 胸引管作为异物插入胸腔内, 本身就会刺激胸膜漏出更多液体, 由于与外界相通, 留置时间越长, 增加感染的几率越大。胸引管会限制与之相接触的局部肺复张, 随呼吸运动刺激肺脏层神经末梢, 使病人为避免疼痛和不适而减小呼吸运动幅度, 增加肺不张的发生。我们在观察24 h内胸引量有减少的趋势后, 即拔除胸引管, 减轻胸引管所带来的胸部并发症。术后活动性出血多发生在术后24 h内, 因此不影响对术后活动性出血的观察。

由于我们术中进行了肋间神经冷冻阻滞, 减少阿片类止痛药的使用, 且早期拔除了胸腔引流管, 鼓励病人术后早期下床活动。一般术后第1天可活动2 h, 第2、3天活动4 h以上。病人早期下床活动有利于刺激肌肉的合成代谢, 避免长期卧床引起的肌肉群的丢失。这一措施使血栓形成、肺部感染等并发症的发生大大减少。

综上所述, FTS可降低手术治疗对病人引起的应激反应, 加速病人的康复, 但其不是单纯追求缩短住院时间, 缩短住院时间和节约医疗资源应建立在高水平的医疗治疗与护理质量基础上, 需要围手术期医生、护士、病人及其家属的通力合作, 让病人一身轻松地出院才是FTS的最终目的。随着FTS理念的不断完善及其应用范围的扩大, 其将为临床治疗带来更大的裨益。我们研究发现:FTS方案用于肺癌外科治疗效果较好。但本研究只是在肺癌治疗中的初步探索, FTS方案中的许多措施应用与肺癌外科还有待进一步改进和完善, 以提高肺癌的外科治疗水平。
